# Cyclooxygenase Inhibition in Sepsis: Is There Life after Death?

**DOI:** 10.1155/2012/696897

**Published:** 2012-05-14

**Authors:** David M. Aronoff

**Affiliations:** Division of Infectious Diseases, Department of Internal Medicine and Department of Microbiology and Immunology, Graduate Program in Immunology, Reproductive Sciences Program, the University of Michigan, Ann Arbor, MI 48109, USA

## Abstract

Prostaglandins are important mediators and modulators of the inflammatory response to infection. The prostaglandins participate in the pathogenesis of hemodynamic collapse, organ failure, and overwhelming inflammation that characterize severe sepsis and shock. In light of this, cyclooxygenase (COX) inhibiting pharmacological agents have been extensively studied for their capacity to ameliorate the aberrant physiological and immune responses during severe sepsis. Animal models of sepsis, using the systemic administration of pathogen-associated molecular patterns (PAMPs) or live pathogens, have been used to examine the effectiveness of COX inhibition as a treatment for severe sepsis. These studies have largely shown beneficial effects on mortality. However, human studies have failed to show clinical utility of COX inhibitor treatment in severely septic patients. Why this approach “worked” in animals but not in humans might reflect differences in the controlled nature of animal investigations compared to human studies. This paper contrasts the impact of COX inhibitors on mortality in animal models of sepsis and human studies of sepsis and examines potential reasons for differences between these two settings.

## 1. Introduction

Sepsis is a major cause of morbidity and mortality worldwide [1], with more than 750,000 cases per year in the United States alone [2]. Despite improvements in diagnosis and therapeutics, there is an ongoing need for better treatments. Sepsis can be defined as a “systemic illness caused by microbial invasion of normally sterile parts of the body” [2], and it can be complicated by organ dysfunction (severe sepsis) or hypotension refractory to volume resuscitation (septic shock) [2].

Research into the fundamental mechanisms of sepsis has historically depended on animal models, with two primary approaches taken to model severe sepsis or septic shock. In one approach, live pathogens are used to cause sepsis. Examples of this approach include (1) inoculating the bloodstream or peritoneal cavity of animals with a single bacterial pathogen, (2) inducing peritonitis via cecal ligation and puncture (CLP), or (3) inoculating the peritoneal cavity of animals with fecal matter. The other approach induces the inflammatory response and complications of sepsis but is not *truly *sepsis since live pathogens are not utilized. In this approach, animals are exposed, usually via intravenous injection, to pathogen-associated molecular patterns (PAMPs) that trigger robust inflammatory responses by activating pathogen recognition receptor-based signaling cascades in the host. Typical PAMPs used to model sepsis include lipopolysaccharide (LPS) from Gram-negative bacteria, peptidoglycan, or mixed PAMPs delivered as inactivated (dead) bacteria.

Animal models of sepsis foster the *in vivo *investigation of signaling cascades that mediate this process. Over the past 50 years, lipid mediators known as prostaglandins (PGs) have garnered significant attention for their roles in mediating the inflammatory and immune response to severe infection. The PGs, oxygenated metabolites of arachidonic acid, are small molecules that have a myriad of roles in regulating pathophysiological responses during sepsis. The synthesis, catabolism, and signaling of PGs have been studied as targets in treating sepsis, particularly when used in combination with antimicrobial agents and supportive care. Early studies targeting PG synthesis in the treatment of sepsis involved inhibiting the cyclooxygenase (COX) enzymes, the first committed enzymatic step in the metabolism of arachidonic acid into bioactive PGs [3, 4].

Despite encouraging results in animal models of sepsis and shock, human pharmacological trials of COX inhibitors have not provided consistent or significantly beneficial findings. The failure of COX inhibitors to significantly improve the outcome of humans with sepsis strongly dampened enthusiasm for targeting PG synthesis for this problem. Why this approach “worked” in animals but not in humans likely reflects important differences in the controlled nature of the investigations involving the former compared to the latter. This unstructured paper explores the impact of COX inhibitors on mortality in animal models of sepsis and human studies of sepsis and examines potential reasons for differences between these two settings. The possibilities for future use of PG-based strategies for treating sepsis are discussed.

## 2. Methods

**Literature Review**

Studies addressing the use of COX inhibitors and nonsteroidal anti-inflammatory drugs (NSAIDs) in animal and human studies of sepsis were identified using the PubMed database (National Library of Medicine, Bethesda, MD). The following search terms were used alone and in combination: “sepsis,” “infection,” “cyclooxygenase,” “prostaglandins,” “PGE_2_,” “receptors,” and “hemodynamic”. Additional references were identified within the bibliographies of PubMed-identified manuscripts. Searches were done for all available publication dates up until January 31, 2012.

Studies were included for analysis if COX inhibitors were administered to animals prior to or in response to the systemic (intravenous or intraperitoneal) administration of PAMPs or if COX inhibitors were administered to animals prior to or in response to a systemic infection model of sepsis. Such sepsis models with live organisms included both *monomicrobial* sepsis models, where a single species of bacteria was administered to animals systemically (intravenous or intraperitoneal) and *polymicrobial* sepsis models, where animals were infected with undefined mixtures of organisms either through the intraperitoneal introduction of stool or through CLP. Studies involving mice genetically deficient for COX isoforms were included as indicated. Human studies were included if COX inhibitors were administered to treat sepsis as defined by the authors of the studies.

The major outcome reviewed from these studies was the effect of COX inhibitors on mortality. Only studies that included data regarding mortality were included for such analyses.

## 3. Results

### 3.1. PAMP Models of Sepsis in Animals

A total of 43 manuscripts were identified that modeled sepsis in animals using the systemic administration of PAMPs to induce a physiological and immunological response similar to clinical sepsis [3–45]. There were 16 publications that did not report mortality data and were excluded from analysis [4–6, 8, 10, 13, 16, 17, 22, 26, 28–31, 36, 41]. Thus, 27 manuscripts were included that provided data regarding mortality after systemic PAMP exposure. Notably, two studies actually conducted studies on two types of species [23, 25], bringing the total number of studies evaluated to 29.

**Figure 1:**
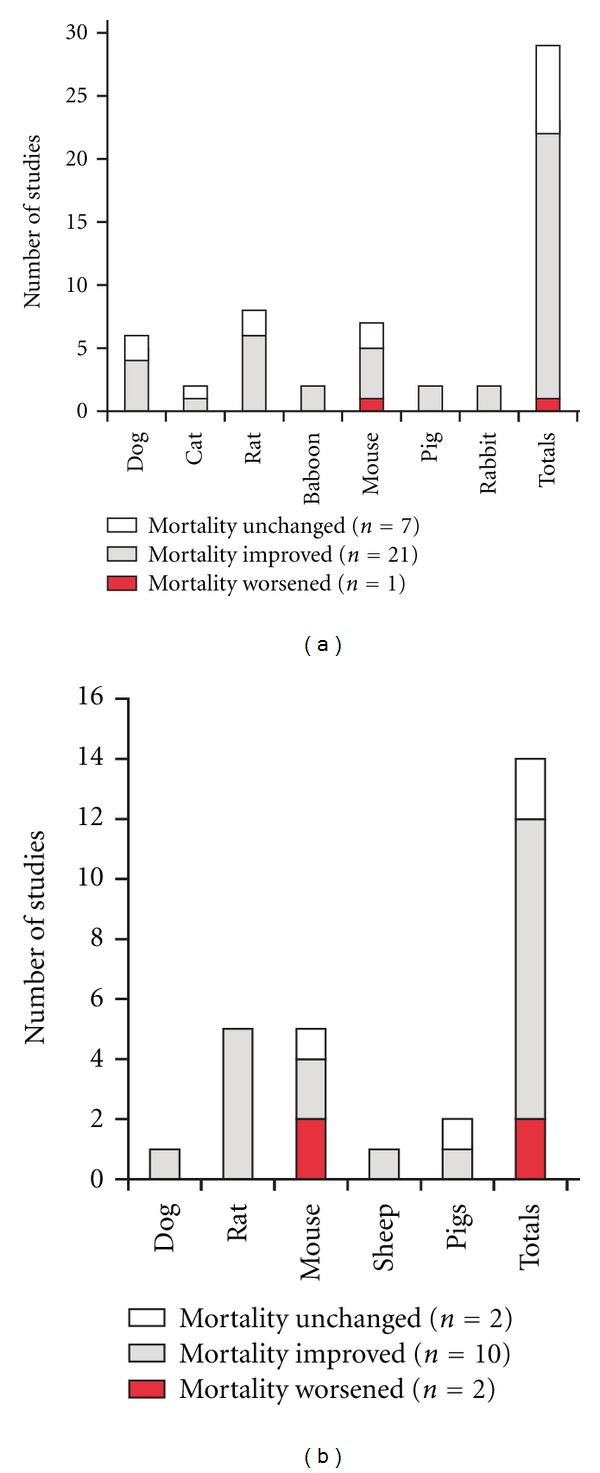
Influence of COX inhibitors on animal models of sepsis. (a) Studies were reviewed for experiments in which mortality was assessed for animals treated with COX inhibitors either before or after the systemic administration of pathogen-associated molecular patterns (generally, lipopolysaccharide). One mouse study (that showed a benefit to survival) included COX-2 knockout animals and not a pharmacological inhibitor. (b) Studies were reviewed for experiments in which mortality was assessed for animals treated with COX inhibitors either before or after the induction of systemic infection (see text for details). One mouse study (that showed a reduction in survival) included COX-2 knockout animals and not a pharmacological inhibitor.

Mortality was assessed in seven different animal species across these studies (Figure 1(a)). Of these, 27 used LPS alone as the sepsis-inducing agent, while one study administered heat-killed *Corynebacterium parvum* prior to LPS [15] and one study used heat-killed Group B *Streptococcus* [34]. The source of the LPS was usually *Escherichia coli* but five studies used *Salmonella* LPS [4, 14, 25, 27, 45]. The PAMPs were usually delivered intravenously, although intraperitoneal approaches were also used. There was great heterogeneity among studies for the COX inhibitor used, the dose employed, whether the drug was administered before or after PAMP exposure, the route of administration of the drugs, and the number of doses of the COX inhibitor. A majority of studies used dual COX-1/COX-2 inhibitors but one study used isoform selective inhibitors [30]. One study was selected that did not use a COX inhibitor but a knockout of the COX-2 gene in mice [12].

The first animal study identified that examined the impact of COX inhibitors on PAMP-induced sepsis was a dog study published in 1962 by Northover and Subramanian [4]. This study was conducted before it was well established that antipyretic and analgesic agents function as COX inhibitors. Thus, the rationale for that work was that salicylate antipyretic agents (sodium salicylate and acetylsalicylic acid) might function as inhibitors of certain protease enzymes thought (at the time) to mediate host cardiovascular responses during bacterial sepsis [4]. While the authors examined actions of these agents on the hemodynamic effects of *Salmonella *LPS, they did not report on the effects of these medications on mortality. Such data were reported, however, in a 1967 study using acetylsalicylic acid in dogs exposed to *E. coli *LPS [3]. That study showed that acetylsalicylic acid significantly reduced the lethality of LPS in the dog model [3].

In total, the studies identified for this paper demonstrated that COX inhibition, and genetic COX-2 deletion [12], improved survival in 21 of 29 studies (72.4%) [3, 7, 9, 11, 12, 14, 18–21, 24, 27, 32, 34, 37, 39, 40, 42, 44, 45], caused no change in survival in 7 studies (24.3%) [15, 23, 33, 38, 43, 46, 47], and reduced survival in a single mouse study (3.4%) [35].

### 3.2. Infectious Animal Models of Sepsis

Seventeen studies were identified in 16 manuscripts that examined the impact of either pharmacological or genetic inhibition of COX enzymes and infection-induced sepsis [46–61]. There were three publications that did not report mortality data and were excluded from analysis [51, 52, 56]. Two studies included data from COX-2 null mice [53, 54].

The models of sepsis used in these studies included three mouse CLP studies [46, 53, 60], three rat studies involving the introduction of feces into the peritoneal cavity [49, 57, 61], two rat studies using systemic infection with live *E. coli* [48, 57], one rat study with systemic infection with live Group B *Streptococcus *[58], two canine studies with systemic infection with live *E. coli* [50, 51], three porcine studies with systemic infection with live Group B *Streptococcus *[47, 56, 59], one mouse study with systemic infection with live *Vibrio vulnificus *[55], one mouse study using live Group A *Streptococcus *[54], and one sheep study of infection with live *E. coli* [48].

Of the 14 studies evaluable, mortality was improved in 10 (71.4%), as depicted in Figure 1(b) [48–50, 54, 57–61].This included one of the two COX-2 knockout mouse studies [54]. Two studies showed no effect on mortality of COX inhibition [46, 47] and two mouse studies revealed increased death in either COX-inhibitor-treated mice [55] or a COX-2 knockout mouse model [53].

### 3.3. Human Studies of Sepsis

Three studies have examined the impact of COX inhibitor therapy on mortality in humans suffering from clinically defined sepsis [62–64], and one study [65] was a subgroup analysis of a larger study [62]. None of the three primary studies showed any positive or negative impact of COX inhibitors on mortality. Two used ibuprofen [62, 63] and one used lornoxicam, a drug relatively more potent against COX-2 than COX-1 [64]. The largest study was conducted by Bernard et al. and randomized 455 subjects to receive ibuprofen 10 mg per kilogram (maximal dose, 800 mg) over a period of 30 to 60 minutes every 6 hours for eight doses or placebo [62]. A similar but much smaller study was conducted by Haupt et al. and randomized 29 patients with sepsis to ibuprofen (600 mg or 800 mg intravenously followed by 800 mg per rectum every six hrs) or placebo [63]. The lornoxicam study by Memiş et al. included 40 subjects with sepsis randomized to receive either lornoxicam (8 mg administered intravenously every 12 hrs for six doses) or placebo [64].

The first human study examining the role of COX inhibitors in sepsis was conducted by Haupt et al. in 1991 [63]. This randomized, double-blind, multicenter study included 29 patients with clinical evidence of severe sepsis (16 were given ibuprofen and 13 were administered placebo). Eight of the ibuprofen-treated patients presented with shock and seven had the acute respiratory distress syndrome (ARDS), while four of the placebo-treated subjects had shock and four had ARDS. Nine patients in the COX inhibitor group died (56%) versus four in the placebo group (31%) (nonsignificant difference) [63].

In 1997, Bernard et al. conducted a larger, multicenter study that also compared ibuprofen with placebo in a randomized, blinded fashion [62]. There were 224 patients in the ibuprofen group and 231 in the placebo treatment arm. Most patients in both groups had two or three organ systems failing at study entry and nearly 50% of subjects had pneumonia in both groups [62]. Shock was present in 65% and 63% of patients in the COX inhibitor group and placebo group, respectively. Thirty-day mortality did not differ significantly in the drug-treatment and placebo groups (37% versus 40%) [62]. Notably, and perhaps relevant to the findings of this study, acetaminophen use was permitted in both arms, and this agent was applied to 44% of subjects in the placebo group (but only 22% in the ibuprofen group) [62].

A substudy of this clinical trial was later published by Arons et al. [65], and it examined hypothermic patients with sepsis who were treated in a randomized, controlled study of ibuprofen at a dose of 10 mg/kg (maximum 800 mg) administered intravenously every 6 hr for eight doses compared with placebo. There were 44 subjects in that study, of which 13 received ibuprofen [65]. A significant reduction in 30-day mortality rate from 90% (18/20 placebo-treated patients) to 54% (13/24 ibuprofen-treated patients) was observed [65].

In 2004, a randomized, placebo-controlled study of lornoxicam was conducted by Memis et al. [64]. Of 40 subjects enrolled, half received the COX inhibitor and half received placebo. Shock was seen on admission in seven patients in the lornoxicam group and eight in the placebo group. The age range of subjects was wide and similar in both groups (for the 40 patients, 19–89 years old). Mortality was 35% in the lornoxicam group and 40% in the placebo group (nonsignificant difference) [64].

## 4. Discussion

Herein we review the impact of COX inhibition on mortality in animal and human models of sepsis. A major finding was that outcomes were better in animal studies than in human studies. While the reasons for this are unclear, it may be important for advancing new treatments for sepsis to more closely explore possible explanations.

When sepsis was modeled in noninfectious, PAMP-driven animal experiments, mortality was improved by COX inhibitors, in 72.4% of studies (Figure 1(a)). These studies spanned seven animal species and only a single mouse study revealed greater lethality when COX inhibitors were used. It is possible that heterogeneity in results related to differences in the dose and microbial source of PAMPs or the type, dose, and route of administration of the COX inhibitors used among studies. On balance, these data suggest that in addition to any effects on host defense mechanisms against live pathogens, COX metabolites increase the mortality resulting from an overwhelming host inflammatory response, possibly due to their importance in systemic vasodilation and renal blood flow [66]. Indeed, several of these studies showed improved hemodynamic parameters in animals treated with a COX inhibitor [3, 5–7, 10, 13, 17, 19, 21–24, 26, 28, 29, 31, 32, 40, 41, 43].

It is also notable that animals with actual infections causing sepsis fared better when COX inhibitors were used (Figure 1(b)). These results could be due to similar mechanisms that protected animals from PAMP exposure. However, in the setting of live infection, another determinant of outcome is the capacity of the host's immune system to eliminate invading pathogens. It is possible that COX inhibitors could influence host immune defense mechanisms since PGs are well known to regulate both innate and adaptive immunity [67–70].

Clearly, animal studies of infection-related sepsis and PAMP-associated inflammation have been able to demonstrate a benefit of COX inhibitor use. Why the lack of effect in human studies? For one, important differences exist in the experimental design between animal studies of sepsis and human clinical trials. It is likely that these differences confound potentially beneficial actions of COX inhibitors in human infection. Animal studies benefit from uniformity among the treated and control groups. Strong similarities (or identicalness) exist in parameters that might impact outcome, including age, gender, genetic background (in mouse studies particularly), general health, rearing environment, commensal microbiota. In addition, the insult (whether infectious or not) is generally highly defined and uniform in animal studies (e.g., all animals will receive the same dose of LPS or the same-sized cecal puncture wound). Animals have also not commonly been treated with antibiotics or other disease-modifying agents during studies of sepsis, which might otherwise alter the results. Lastly, in many animal studies of sepsis, COX inhibitors were given *prior *to the onset of overwhelming inflammation or infection, though studies giving the medication *after *the onset of sepsis have generally concurred [48, 57, 59, 71].

In stark contrast to the highly controlled animal studies, human clinical studies suffer from variability in almost every measurable aspect. Subjects with sepsis are not uniform in age, gender, comorbidities (or the presence of immunosuppression), the cause of sepsis (a major difference), the timing of therapy relative to the onset of symptoms, and so forth. Another major difference is that human subjects receive supportive care beyond that generally administered to animals, including intravenous fluids, vasopressive agents, mechanical ventilation, blood products, surgery, and (perhaps most importantly) antibiotics. Thus, the lack of impact of COX inhibitors in human studies may be due to a lack of similarity within and between cases and controls. It is also possible that the incremental effect (whether beneficial or detrimental) of COX inhibitors is too small to measure reliably when other supportive therapies are making more significant impacts on patient recovery.

The lack of clear benefit for COX inhibitors in human studies of sepsis begs the question of whether there is any worth in continuing to investigate PG synthesis and signaling cascades as targets for sepsis treatment. Given the risk of “throwing out the baby with the bathwater,” it is important to determine whether the wealth of animal studies has provided a clue to novel therapies that remains undiscovered. For example, COX inhibitors might be most beneficial if given early in sepsis when patients are otherwise relatively robust: young, free of comorbidities, and so forth. Unfortunately such a patient population is a minority in the world of sepsis.

An alternative notion to explain the lack of benefit of COX inhibitors in human sepsis is that specific prostanoid molecules might need to be targeted, as opposed to blocking the most proximal committed step in PG synthesis. Perhaps in human cases of sepsis some PGs are helpful and others maladaptive. This approach has been taken in both animal and human studies [72]. For example, inhibiting the synthesis of thromboxane [73, 74] has been attempted in small human studies of sepsis and acute respiratory distress syndrome with disappointing results. Animal models have been used more extensively to study select COX-derived eicosanoids [28, 75]. An example is provided by the molecule PGE_2_, whose synthesis and signaling is increasingly being investigated as a target for immunotherapy in severe infections [54, 76–78].

In summary, myriad highly controlled animal models of sepsis provide a strong rationale for the targeting of PGs in the treatment of sepsis. However, the relatively small number of human studies has failed to support this approach. While the reasons for these differences are unclear, future studies are warranted to identify either particular human populations who might benefit from COX inhibitor treatment during sepsis or to identify particular prostanoids whose synthesis or signaling pathways can be specifically targeted during sepsis management.
